# Introducing cross-cultural education in palliative care: focus groups with experts on practical strategies

**DOI:** 10.1186/s12904-020-00678-y

**Published:** 2020-11-10

**Authors:** Imane Semlali, Emmanuel Tamches, Pascal Singy, Orest Weber

**Affiliations:** 1 Liaison Psychiatry Service, Department of Psychiatry, Lausanne University Hospital, Av. de Beaumont 23, 1011 Lausanne, Switzerland; 2 Palliative & Supportive Care Service, Lausanne University Hospital, Av. Pierre-Decker 5, 1011 Lausanne, Switzerland

**Keywords:** Cultural competence, Cultural sensitivity, Palliative care, Cross-cultural training, Communication

## Abstract

**Background:**

The linguistic and cultural diversity found in European societies creates specific challenges to palliative care clinicians. Patients’ heterogeneous habits, beliefs and social situations, and in many cases language barriers, add complexity to clinicians’ work. Cross-cultural teaching helps palliative care specialists deal with issues that arise from such diversity. This study aimed to provide interested educators and decision makers with ideas for how to implement cross-cultural training in palliative care.

**Methods:**

We conducted four focus groups in French- and Italian-speaking Switzerland. All groups consisted of a mix of experts in palliative care and/or cross-cultural teaching. The interdisciplinary research team submitted the data for thematic content analysis.

**Results:**

Focus-group participants saw a clear need for courses addressing cross-cultural issues in end-of-life care, including in medical disciplines outside of palliative care (e.g. geriatrics, oncology, intensive care). We found that these courses should be embedded in existing training offerings and should appear at all stages of curricula for end-of-life specialists. Two trends emerged related to course content. One focuses on clinicians’ acquisition of cultural expertise and tools allowing them to deal with complex situations on their own; the other stresses the importance of clinicians’ reflections and learning to collaborate with other professionals in complex situations. These trends evoke recent debates in the literature: the quest for expertise and tools is related to traditional twentieth century work on cross-cultural competence, whereas reflection and collaboration are central to more recent research that promotes cultural sensitivity and humility in clinicians.

**Conclusion:**

This study offers new insights into cross-cultural courses in palliative and end-of-life care. Basic knowledge on culture in medicine, variable practices related to death and dying, communication techniques, self-reflection on cultural references and aptitude for interprofessional collaboration are central to preparing clinicians in end-of-life settings to work with linguistically and culturally diverse patients.

## Background

In many Western countries, palliative care has evolved quickly over the past decades as growing numbers of individuals face complex, chronic and terminal diseases [1, 2]. Interpersonal communication is a central aspect of palliative care, which aims to address patients’ physical, psychological, social and spiritual needs at end of life and in severe illness [3]. Communication skills are therefore essential in palliative care, and a large part of training in palliative medicine is aimed at the acquisition of these skills [4].

When clinicians, patients and relatives belong to different linguistic and cultural groups, clinical encounters in palliative care can involve major challenges. These can be related to language barriers, a lack of specific institutional resources (e.g. written material in several languages, interpreters), patients’ and relatives’ highly divergent expectations and perceptions, and risks of stereotyping [5, 6]. When these challenges are not addressed efficiently, medical systems cannot guarantee equitable care [7, 8].

In Switzerland, increasing immigration has forced health care systems to adapt their practices to respond to patients’ needs [9]. Specific policies have been enacted to address the challenges that have resulted. This includes Swiss Hospitals for Equity, a project working at the organizational, structural and clinical levels to address health disparities and guarantee equal access to care and equal treatment [10]. At the organizational level, the project federates 12 hospitals which evaluate their organization according to a self-assessment tool and subscribe to a charter. Access to interpreters, availability of social workers for vulnerable patients are among the criteria of assessment. The project also promotes cross-cultural competence and cultural sensitivity training, which involves a mix of knowledge, skills and attitudes that help clinicians provide quality care to culturally diverse patients [11, 12].

Hospice care has a long tradition in Switzerland, but outpatient, mobile palliative care offers and bed units are still expanding. Indeed, the global offers remains discrepant among the national territory. Central issues in palliative care are the promotion of a palliative paradigm –in hospitals and in the general population– and of a patient and family-centered approaches with seriously or terminally ill patients [13]. In palliative care, cultural consideration is essential around issues such as decision-making and symptom management [14]. Cross-cultural competence and sensitivity are especially important for clinicians because “cultural beliefs, values, and experiences shape each patient’s definition of a ‘good death’” [14, 15]. In Switzerland, training offerings for palliative care professionals rarely present aspects related to cross-cultural competence. Educators thus need practice-based insights in order to implement specific cross-cultural content into palliative care curricula.

This paper presents ideas derived from a focus-group study with key actors in the fields of palliative care and cross-cultural clinical work about the integration of cross-cultural content into palliative care training. Three research questions oriented the study: (1) What are the goals and topics for cross-cultural training in end-of-life care? (2) How can the organization of the current training system be improved? (3) Which teaching methods should be applied? The study is part of a bigger project aiming to deliver recommendations to educators, training managers and institutional decision makers.

## Methods

We conducted 4 focus groups in the French- and Italian-speaking parts of Switzerland. Each encounter was located in a different region (Geneva, Lausanne, Neuchâtel, Bellinzona). We gathered data by stimulating discussion and joint reflection about cross-cultural education in palliative care curricula with experts in palliative care and experts in cross-cultural training. Focus groups were an ideal research tool for our study. Focus groups facilitate the expression of ideas and experiences that might be left underdeveloped in an individual interview, and they illuminate participants’ perspectives through debate and conversation within the group [16]. For each group we recruited clinicians, training managers and teachers from palliative and/or cross-cultural fields in order to guarantee a diversity of opinions and perspectives. We estimated that 4 focus groups were enough to achieve data saturation. Groups had 6–7 participants, enough to gain a variety of perspectives without becoming too disorderly [17]. Table 1 presents information on participants.

**Table 1:** Focus-group participants’ information

Focus groups	Attendees	Status
1	6	Physician, unit leader, teacher (palliative care) Physician, teacher (palliative care) Psychologist, specialist in intercultural psychology Medical anthropologist, teacher in cross-cultural competence Physician, head of migrant health program Chaplain
2	7	Physician, service leader, teacher (palliative care) Physician, (palliative care/geriatrics) Psychiatrist, migrant care unit Anthropologist, specialist in palliative care Senior nurse, training manager (palliative care) Physician, head of Psychiatry, specialist in cross-cultural psychiatry Chaplain
3	7	Physician, unit leader (palliative care) Physician, service leader, teacher (palliative care/oncology) Senior nurse, teacher (palliative care) Sociologist, migration specialist Senior nurse, training manager (palliative care) Doctor in social science, migration specialist Theologist, specialist in religious minority
4	6	Physician, training manager (oncology/palliative care) Physician, service leader (palliative care) Senior nurse, training manager (palliative care, cross-cultural clinic) Senior nurse, training manager (geriatrics) Senior nurse, cross-cultural clinic specialist Chaplain

The participants received oral and written information about the study and signed a consent form confirming their voluntary participation. The focus groups lasted between 85 and 91 min, including an introduction that covered two points in order for participants to have a common information base:

1. A definition of cross-cultural competence and sensitivity as “a set of competences and attitudes allowing clinicians to interact efficiently with socially, linguistically and culturally diverse patients” [18].
2. An inventory of actual palliative care training in Switzerland.

The discussion was organized around 6 questions:

1. In your opinion, what are the priorities for cross-cultural training in specialized palliative care?
2. What type of training activities do you see as useful?
3. Where, in the training program of physicians who specialize in palliative care, would you imagine the introduction of cross-cultural training?
4. Where, in the training program of nurses who specialize in palliative care, would you imagine the introduction of cross-cultural training?
5. Where, in the training program of other professionals (e.g. care assistants, social workers, psychologists) who specialize in palliative care, would you imagine the introduction of cross-cultural training?
6. What could be promptly and easily changed or improved?

Follow-up questions helped participants think practically about strategies to improve the field of palliative care training and clarified any information gathered from participants where needed.

The focus groups were conducted between October 2018 and January 2019 by two members of the research team. We made full audio recordings that we subsequently transcribed verbatim and analysed using thematic analysis, according to Rabiee [19] and Meuser and Nagel [20]. The transcripts were divided into units of meaning that were given one or more thematic codes. These codes are subsequently grouped into categories and, at a higher level, into themes. IS, OW and ET performed the primary coding, and IS and OW reapplied the thematic grid to the entire corpus and adapted it in collaboration with ET. All authors discussed the final description of the content. The thematic categories presented here are items from the final thematic grid.

## Results

In all focus groups, there was lively discussion. Participants all agreed that social, cultural and linguistic diversity in patients creates many challenges that clinicians must be able to handle thanks to cross-cultural clinical education. Table 2 presents all the content categories and themes, grouped according to our research questions. Many categories were mentioned several times in the focus groups. The second column of Table 2 contains the number of hits (i.e. mentions) for each category.

**Table 2:** Categories of content of the focus-group transcripts

**Goals and topics of training**	**Hits**
Background knowledge:	63
Introduction into culture and health	8
Range of patients’ and relatives’ expectations/perceptions	34
Basic information for clinical practice	21
Communication tools/techniques for patient-centred care:	48
Investigating patient’s reality/projects/needs	16
Handling inhibitions	6
Interpreting forms of cultural expressions	4
Managing tensions between patient and health care system	4
Establishing rapport	2
Displaying commitment in communication	2
Embracing a human attitude (savoir-être)	2
Overcoming language barriers	2
Listening	1
Negotiating institutional rules	1
Managing non-verbal communication	1
Explaining palliative care to culturally diverse patients	1
Collaboration:	10
Interprofessional collaboration	3
Collaboration with relatives	7
Reflection on culture and values:	26
Cultural diversity among clinicians	8
Self-reflection (impact of clinicians’ own culture and stereotypes)	8
Tensions that result from cultural difference	6
**Organization of the training system**	**Hits**
Integration of transcultural training into existing training programs	43
Undergraduate education	14
Postgraduate education	9
Interprofessional continuing education	20
In palliative care	16
In cross-cultural care	4
Intervention throughout the training curriculum	6
Extensions beyond specialised palliative care	13
Cross-cultural education for related medical fields	8
Introduction on palliative care for interpreters and experts in cross-cultural care	3
Cross-cultural education for non-specialized palliative care	2
Interprofessional training (e.g. Congresses, Symposia, Seminars)	14
Differentiation of training according to disciplines	9
Risks	8
Clinicians’ lack of time for continuing education	4
Low motivation because of lack of time spent with patients	2
Overfilled palliative care curricula	1
Overfilled undergraduate medical curricula	1
**Teaching methods**	**Hits**
Face-to-face theoretical teaching (Lectures)	7
Practical training	19
Workshops with case discussions	6
Role plays	6
Immersive experiences	6
Comparative approaches of two cultures	1
Interprofessional work experience	4
Coaching	17
Supervisions, colloquia	11
Mentoring	4
Exemplarity	2
Independent learning	2
Training tools	5
Toolbox (leaflets, catalogues)	2
Platforms health and spirituality’	1
Telecommunication tools	1
E-learning platforms	1

### What are the goals and topics for cross-cultural training in end-of-life care?

The participants’ discourse about the goals and topics of cross-cultural training was abundant and thematically rich. It covered four main areas: background knowledge, communication tools and techniques, ability to collaborate, and self-reflection.

### Background knowledge

According to participants, clinicians need to acquire a definition of culture as a multilayered, flexible construct that affects health and clinical interactions and is not limited to the situation of migrants. For example, clinicians and hospitals have a ‘culture’ as well. However, several participants were quick to emphasize the importance of culturally based religious and spiritual aspects in palliative care, which are particularly challenging for the field.

“*There should be some elements about culture and society, and how [those] influences individuals from the moment they are born*.” (Focus group 2, Participant 3)

Experts further suggested that, with such a comprehensive definition of culture, clinicians in cross-cultural training should be sensitised to the existence of a broad range of patients’ and relatives’ expectations and perceptions. In this context, this means emphasizing the tremendous diversity of ways to accompany dying persons and rituals around dying and death.

*“It is not describing practices in each community that matters, but being aware of the « range of variation » (…) the diversity of expectations, the diversity of beliefs, or the diversity of values that can exist”* (FG1, P3)

Many focus-group participants recommended the presentation of practical information as an additional aspect of the transfer of background knowledge. This encompasses the provision of lists of key informants and experts of cross-cultural issues, which palliative care clinicians can rely on. Several palliative care specialists also mentioned the possibility of presenting checklists of death-related rituals and information sheets on major approaches to death and dying by the religious and cultural groups that clinicians are likely to face. The frequent traumatic experiences of those who went through forced migration should also be tackled.

### Communication tools and techniques for patient-centred car

In all the focus groups, participants insisted that culturally sensitive care is, above all, patient-centred care. Some of the experts in cross-cultural and palliative care criticized checklist approaches as leading to the cultural stereotyping of patients. They would prefer that cross-cultural training focus on the acquisition of specific communication tools and techniques to support patient-centred care with a diverse population. These tools and techniques must be developed with a thorough investigation of patients’ individual realities, goals and needs.

*“I think that when we go towards static packs of culture, we risk to fall into stereotypes. So to me, it is right from the start that the clinician must try to know the patient, and understands him as a unique character: what are his wishes? His will? But we must not in any way say that we ignore the culture of others, their habits and religious contexts.”* (FG4, P5)

A few participants mentioned that asking questions is not always enough, and that clinicians need help in order to interpret correctly patients’ culturally based expressions of concerns and needs. Experts also mentioned that clinicians must learn how to overcome obstacles in clinical encounters related to inhibitions (e.g. taboo, intimacy) and tensions over institutional rules (e.g. permitted number of visitors). This topic was identified as closely connected to courses focused on rapport and commitment in care, which experts also recommended.

### Collaboration

Clinicians’ abilities to collaborate with other persons for the sake of patients’ care also received attention on the part of focus-group participants. They mostly mentioned a need for courses on collaboration with relatives in the context of potential ethical dilemmas about decision-making and disclosure. In contrast, only a few participants pointed to a need for education in interprofessional or interdisciplinary communication. A key message of participants was that clinicians should learn to identify situations that require the help of additional professionals who have a variety of backgrounds (e.g. spiritual leaders, medical anthropologists).

*“First, we have to know that maybe in this kind of situation, we have to look for help”* (FG1, P4)

Participants promoted collaboration with interpreters only twice. They did mention that because of the responsibility placed on interpreters with their translation of delicate information to patients and relatives, they should also have access to training about end-of-life health care issues.

*“They help clinicians by giving cultural information, but they also need to receive training, because they are invested with a responsibility that is not without consequences*” (FG3, P7)

### Reflection on culture and values

Numerous experts stated that cross-cultural training in palliative care must engage participants in reflective activities about their own cultural background and preconceptions, as well as any possible stereotypes they hold.

*“It’s a kind of awareness of the values caregivers bring to the situation…institutional, professional, and personal. Because if we’re not aware of our own norms, expectations, values, etc, it’s a little difficult”* (FG1, P4)

Such awareness is especially important in increasingly multiethnic and multilingual care teams. The diversity of values in these teams is at once a source of potential tension and a significant resource.

### How can the organization of the current training system be improved?

All the experts agreed on the need to insert cross-cultural content into already existing training courses on larger topics whenever possible, to avoid any unnecessary redundancy across multiple training offerings. Aspects of cross-cultural competence and sensitivity should be distributed throughout curricula (e.g. undergraduate, postgraduate, continuing education) of all kinds for professionals working in palliative care. Fundamentals of cross-cultural education could be taught at the undergraduate level, whereas specific content related to diversity at the end of life could be introduced at later stages.

With regard to our research focus on specialised palliative care, many participants stressed the importance of organising cross-cultural training in a way that expands beyond the limits of this narrow field. They referred to general palliative care (i.e. carried out by family doctors, homecare nurses, etc.), and also to clinicians from other medical fields caring for end-of-life patients. As an additional suggestion, they promoted the introduction of end-of-life care into curricula for community interpreters and cross-cultural care experts (e.g. chaplains and spiritual leaders or directors, medical anthropologists).

*“We (palliative care unit) have our 2 days and a half of introduction into palliative care, I think we should implement something about culture there, and you (talking to an expert in cross-cultural care) could implement something about palliative care in your course, and eventually that’s how we can meet each other.”* (FG1, P1)

Courses in cross-cultural competence and sensitivity for clinicians specialising in palliative care should involve a wide variety of professions whenever possible. In order to support and encourage professional diversity, several interviewees reported a need for events such as congresses, symposia and seminars that focused on culture, language and care. However, there are also aspects of competence and sensitivity that are particularly important for a specific profession, such as doctors having to break bad news to patients. These can be included in profession-specific curricula or into events for specific professions.

According to our focus groups, in practice, implementing cross-cultural courses may be difficult. The main obstacles reported were overfilled schedules in medical schools and in palliative care curricula. Some clinicians, mainly doctors, may also lack the time to join multidisciplinary training events.

*“Theoretically these continuing training courses are for everyone and then we see that doctors do not come”* (FG1, P4)

As some experts stated, working conditions in palliative care may also hamper clinicians’ motivation for cross-cultural courses: when clinicians already lack the time requested of them to provide genuinely patient-centred care in their everyday practice (e.g. because of late referrals to palliative care), they do not see the point of attending such trainings.

### Which teaching methods should be applied?

As far as teaching methods are concerned, they are closely related with the main training goals mentioned earlier. Focus-group participants suggested face-to-face teaching for the transmission of basic knowledge on culture and clinical practice with migrants.

*“I think it’s important to [include] courses, to give basic knowledge of cross-cultural concepts*” (FG3, P6)

For the acquisition of patient-centred communication techniques, self-reflection and collaborative skills, they suggested methods such as workshops with case-study discussions, role plays and immersive experiences (ex. visit to residences of individuals seeking asylum).

The focus groups also brought to light a few quite original didactic approaches, in particular several forms of coaching by expert professionals. These included supervision and colloquia, but also one-to-one mentoring by an experienced colleague.

*One idea is coaching for other professionals. For me coaching is a really interesting pedagogical method.* (FG2, P1)

An additional suggestion was to provide resources for clinicians who are eager to acquire knowledge independently, such as leaflets, catalogues and online tools.

## Discussion

This study used focus groups to collect key information about cross-cultural training initiatives in palliative care. It was carried out at the interface between palliative care specialists and cross-cultural care and teaching. Many major points of agreement exist in the discourses of these two areas, and those points often echo elements of the existing literature. For example, our focus-group participants pointed out that attending to the specific needs of culturally diverse patients requires clinicians to have specific knowledge, skills and attitudes, as described in mainstream literature on cultural competence and sensitivity [18, 21, 22].

The information gathered from the focus groups also corroborates that there is a strong pre-existing sensitivity to diversity issues in palliative care within a genuinely patient-centred clinical work [23, 24]. In addition, the palliative care discipline has a strong culture of interprofessional collaboration. This is perceptible when experts call for courses in which all kinds of palliative care clinicians receive joint cross-cultural education. However, there appears to be only moderate interest in learning how to collaborate with interpreters and other specialists in cross-cultural clinical work. This may be partly due to limited access to interpreters in some palliative care systems found in French- and Italian-speaking Switzerland. But it may also be due to the health care professionals’ own ambivalence toward interpreter-mediated patient consultations, associated with their fear of losing control [25, 26]. In palliative care, interpreters do however experience as difficult their sudden immersion in complex end-of-life situations [27]. Palliative care clinicians should be able to brief them before their first common consultation about the case and its communicational, emotional and relational challenges.

Regarding cross-cultural care and teaching, our focus groups reflected a strong motivation among participants to convey useful knowledge, skills and attitudes to (future) palliative care and end-of-life specialists. As do many other clinicians [28, 29], some palliative care experts, especially the physicians, seem attracted to checklists and tools for cross-cultural clinical work and communication. They refer mainly to concise information about religious and traditional beliefs and practices in migrants’ countries of origin. However, cross-cultural education specialists and some palliative clinicians insist on the risks of stereotyping with minority patients. In their opinion, this may happen if cross-cultural education presents ready-made categories of cultural practices to audiences.

The debate about the usefulness and risks of transmitting detailed checklists of information about cultures to clinicians reflects different paradigms within the literature on the handling of cultural issues in clinical work [22, 30, 31]. Several recent publications have emphasized that simplistic or categorical forms of teaching about culture may generate problematic feelings of certainty in participants. As a result, instead of asking for help and questioning patients and relatives, clinicians may try to manage complex cultural dilemmas on their own, by making reference to a false sense of their own cultural competence [15, 32, 33]. This is one of the major reasons several contemporary authors have preferred concepts of cultural sensitivity, humility and/or uncertainty [22, 30, 31] to “cultural competence.” For them, the objective is not to have encyclopaedic knowledge about culture or to apply formulaic guidelines for communication with minority patients; rather, it is to guarantee that *all* patients receive culturally appropriate and safe care [22, 34]. This means that professionals and institutions must carry out clinical encounters with adequate sensitivity to patients’ social, political, linguistic, economic and spiritual circumstances; and they should empower patients through decision-making processes [22, 35]. A major challenge is Advance Care Planning in a context of potentially diverging cultural views on patient autonomy and of strong social asymmetries between patients and medical institutions when migrants are involved.

The concerns underlying the concepts of cultural sensitivity or humility and the recent criticism of mainstream concepts of cultural competence are of prime importance for achieving positive effects through cross-cultural teaching. This leads us to a few suggestions for the main themes of cross-cultural courses, as mentioned by focus-group participants. We recommend that course offerings start with fundamental aspects of cultural and linguistic diversity in end-of-life care, which are anchored in a multifaceted and flexible definition of culture, and take into account the institutional culture of health services, social class, power relations and so on [36, 37]. As many participants in our study stated, clinicians should become aware of the range of practices and expectations that they are likely to encounter with patients and relatives, and they should improve their ability to proactively investigate which practices and expectations are relevant in each case and then adapt their practice to those. Self-reflection is a very important aspect to include in cross-cultural training [31, 33]. Course participants must examine their own cultural background both as a human being and as a health professional. Their awareness of the impact of ethnocentric and socioeconomic institutional rules, beliefs, practices and values on clinical practice has to be raised [38]. Even though few participants mentioned this topic, clinicians receiving training in cultural competence must learn about the existence and roles of other professionals who can help them in complex cultural and linguistic situations and how to collaborate with those professionals [25, 27].

Educators who organize and implement cross-cultural teaching in palliative and end-of-life care should be aware that participants and decision makers in these fields are attracted to information conveyed in checklists and as concise communication tools. Therefore teachers should explain to students the risk associated with presenting and learning information in this way. Moreover, careful handling of cross-cultural contents should by no means exclude very practice-oriented courses that address the difficulties clinicians face in their everyday working environment and avoid generalities and theory.

Discussions of clinical case studies and educational films, as well as role plays, are common ways to ensure a close connection with actual clinical work. By focusing on individual patients and families, these discussions also help to show how to deal with cultural diversity without prioritizing one culture over another: individuals have complex beliefs and ways of communicating, and these may sometimes be contradictory (e.g. a Catholic woman might identify as a feminist and support abortion rights but let a man speak on her behalf in medical consultations). Discussions can thus lead participants to adopt a humble stance toward diversity and simultaneously test useful practices for investigating it (e.g. questions such as outlined by Kleinman et al. [37]). For the acquisition of relational and communication aptitudes and attitude, focus-group participants also suggested original techniques, such as mentoring by more experienced and well-trained colleagues. In order to endorse mentor roles in this kind of teaching, a growing number of senior palliative clinicians might become referent persons in cross-cultural care [28].

As far as the organization of cross-cultural education and palliative and end-of-life care is concerned, the experts in our focus groups see a risk of counterproductive compartmentalization. The careful introduction of elements of cross-cultural teaching into globally applicable curricula for clinicians working in palliative and end-of-life care is therefore a major concern. Courses on cross-cultural aspects for clinicians should start in undergraduate education, so that professionals training in palliative care already have basic knowledge when they join this specialty. Later, postgraduate and continuing education training about diversity at the end of life should target palliative care specialists as well as other specialists dealing with death and dying, such as nurses and doctors involved in general palliative care, oncologists, intensivists, geriatricians, and so on.

A rather marginal but very important suggestion from our focus-group participants was the development of courses on end-of-life issues for interpreters and chaplains of different religious groups. A recent literature review emphasized the importance of these issues for interpreters [25, 27]. Palliative care specialists and other experts of end-of-life care should be part of the teaching staff for training courses designed for interpreters.

Our results provide a starting point for further reflection on cross-cultural education in end-of-life care and for the elaboration, in different national and regional contexts, of plans of action on this topic. In each context, our work can help define cross-cultural teaching objectives at different curricular stages for clinicians who choose to work in end-of-life care and those objectives can be tied into educational venues and teaching methods. In order to achieve this goal, collaboration will be necessary among experts in palliative and end-of-life communication, general communication skills training and cross-cultural training [39]. In the Swiss context, two authors of this publication are currently engaged in a follow-up project aiming to elaborate a plan of action on behalf of Switzerland’s Federal Office of Public Health (FOPH).

There are some limitations to our study. Our focus on only palliative care excludes information from other fields that work in end-of-life care (e.g. oncology, geriatrics). Among the professions that contribute to palliative care, we invited mainly physicians, nurses and chaplains to participate in our focus groups. We were not able to incorporate other essential professions (e.g. physiotherapists, occupational therapist, social workers), and their point of view would also be of interest to our study. The study was also mainly centred on the hospital and medicine, leaving outside of reach important groups of potential informants, such as community interpreters and representatives of migrant community organisations. We will hopefully be able to incorporate their contributions in future work on this topic.

## Conclusion

Our study corroborates a need for courses on cross-cultural issues for professionals who are involved in end-of-life care. The focus groups stressed the importance of integrating such content into existing training offerings and ensuring that basic knowledge is acquired during undergraduate training, even before professionals decide to specialize in end-of-life-related disciplines. In postgraduate and continuing education, joint learning among different professions is promoted. Two trends emerge related to course content: the acquisition of cultural expertise and tools for professionals to deal with complex situations on their own, and the importance of clinicians’ reflecting and learning how to collaborate with other professionals in complex situations. These trends evoke recent debates in the literature: the quest for expertise and tools is related to traditional mainstream work on cultural competence, and reflection and collaboration are central to more recent research that promotes cultural sensitivity and humility in clinicians. Basic knowledge on culture in medicine, variable practices related to death and dying, communication techniques, self-reflection on cultural references and aptitude for interprofessional collaboration are therefore very important in preparing clinicians working in end-of-life settings to take care of linguistically and culturally diverse patients.

## Data Availability

The datasets generated and analysed during the current study are not publicly available to respect participants’ anonymity, but are available from the corresponding author on reasonable request.

## Abbreviations

FG: Focus group
P: Participant
FOPH: Federal Office of Public Health
